# Vascular Access in Older Adults Approaching Kidney Failure: Is There a Single Right Time?

**DOI:** 10.34067/KID.0000001373

**Published:** 2026-09-24

**Authors:** Todd Robinson, Mariana Murea

**Affiliations:** Department of Internal Medicine, Section on Nephrology, Wake Forest School of Medicine, Winston-Salem, North Carolina

**Keywords:** access blood flow, arteriovenous fistula, arteriovenous graft, hemodialysis access, vascular access

Guideline-defined on-time arteriovenous fistula (AVF) creation, *i.e*., within 6 months before dialysis initiation and with a functioning AVF at hemodialysis start, has implicitly promoted two interconnected notions: a right timing for AVF creation exists, and this discrete window can be predicted with reasonable precision.^1^ In reality, this construct is much less secure, particularly in older adults with advanced CKD, in whom competing risks such as mortality, frailty, and functional decline often rival or outpace kidney disease progression.^2^ In this issue of *Kidney360*, Heggen *et al*., present a secondary analysis of the European QUALity Study on treatment in advanced CKD (EQUAL) vascular access surgery timing (VAST) cohort that adds further insight into the vexing question of when AVF creation is right.^3^

The EQUAL study is a prospective, multicenter European cohort designed to characterize the clinical course of older adults with advanced CKD. Among 1736 participants, 332 patients who underwent AVF creation and later initiated hemodialysis were included in the VAST analysis.^3^ The authors categorized AVF timing as early, on time, or late according to the interval between AVF creation and dialysis initiation and whether dialysis was started with a functioning AVF rather than a central venous catheter (CVC). Early timing—defined as AVF creation without dialysis initiation within 6 months—occurred in 42% of patients; on-time creation, defined as initiation of dialysis with a functioning AVF within 6 months, occurred in only 32%; and late creation, defined as initiation with a CVC within 6 months of AVF placement, occurred in 26%.^3^ Yet, nearly 90% of patients ultimately initiated dialysis within 3 years.

The report by Heggen *et al*. underscores a reality of clinical practice: judged by the definition of on-time AVF creation, clinicians can identify patients likely to require dialysis in the foreseeable future, but predicting dialysis initiation timing in month-scale window is achieved in only roughly one-third of instances. Disease trajectories in advanced CKD are highly heterogeneous, especially in older adults. Periods of prolonged stability may be followed by abrupt decline, often precipitated by AKI, cardiovascular events, infection, or hospitalization. Such intercurrent events can compress decision making such that the right window of preemptive AVF preparation is no longer feasible. At the other end of the spectrum, patients referred early for access creation may never progress at the anticipated rate. Other studies have shown that a substantial proportion of older adults undergoing predialysis arteriovenous access creation either initiate dialysis much later than anticipated or never use the arteriovenous access at all.^4^ And most importantly, not all apparent mistiming reflects a mistimed decision. Because primary maturation failure remains common in older adults, some patients classified as late may have undergone access creation at an indicated moment, only to have the fistula fail to become usable in time. The problem therefore is not simply one of poor forecasting; it is that the on-time vascular access definition is applied to a biologically and clinically dynamic process as if it were static, and to a surgical intervention as if its success were guaranteed and unidirectional.

The complexity of timing in AVF surgical creation is compounded by the possibility that early AVF creation may itself favorably modify kidney disease progression. A few observational studies have reported slower eGFR decline following AVF placement.^5^ These associations are difficult to interpret and possibly reflect selection bias, confounding, or immortal time bias, remaining uncertain whether AVF creation has an independent beneficial effect on eGFR stabilization through hemodynamic or biologic effects.^6^ Thus, AVF placement should not be viewed as a disease-modifying intervention but rather as a preparatory step contingent on individualized risk and goals.

Patient preference adds another critical dimension to this process.^7^ The recently reported clinical trial by Quinn *et al*., conducted in Canada and Australia, included patients 55 years and older who started chronic hemodialysis with a CVC and underscored substantial reluctance among patients to accept randomization between AVF and CVC, with many preferring to continue CVC-based hemodialysis.^8^ Such preferences convey procedural burden, fear of surgery, uncertainty about future dialysis need, or difficulty accepting the reality of kidney failure. These preferences are rarely captured in either prospective or retrospective studies, yet they contribute to what is often perceived as imprecision in clinician prediction of dialysis initiation. In some cases, an apparent failure of prognostication may instead reflect patient ambivalence or deferral of recommended arteriovenous access creation. Vascular access selection is therefore not solely a technical or prognostic decision; it is also shaped by patient values, treatment goals, and quality-of-life considerations.

The EQUAL-VAST study has notable strengths, including its prospective design, multinational cohort, and focus on older adults, a population often underrepresented in vascular access research.^3^ Its limitations include an observational design that precludes casual inference, exclusion of patients without nephrology follow-up, and limited data on actual AVF use and reinterventions. Even so, the study reinforces two key points. First, treating vascular access timing as a one-time decision creates a dual risk: premature intervention in some patients and delayed preparation in others. Second, judging the quality of access preparation against rigid time-based thresholds may not be well suited to a process that is inherently uncertain and continuously evolving.

This is where prediction tools may help refine clinical judgment. Heggen *et al*. propose incorporating the Kidney Failure Risk Equation (KFRE) into access planning, suggesting that a ≥40% 2-year kidney failure risk threshold may better balance the risks of premature AVF creation against those of prolonged catheter dependence.^9^ Supporting this approach, a population-based study from British Columbia found that adding KFRE to standard eGFR-based referral criteria increased the proportion of patients who initiated hemodialysis with an AVF or graft from 49% to 58%, while reducing too-early access creation (defined as AVF/graft placement not followed by hemodialysis initiation within 2 years) from 31% to 18%.^10^ These findings suggest that a risk-based framework may align access creation more closely with actual dialysis need than eGFR thresholds alone.

Still, prediction tools ought not be interpreted in isolation. In older adults with advanced CKD, access planning is better understood as the integration of baseline vulnerability, disease trajectory, and competing risk, all viewed through the lens of patient goals and decisional readiness (Figure 1). Baseline vulnerability includes age, frailty, comorbidity burden, and vascular anatomy. Disease trajectory reflects eGFR slope, prior AKI, and overall clinical stability. Competing risk incorporates KFRE alongside risks of mortality and functional decline. These domains are not fixed. Acute intercurrent illness can rapidly alter the expected course and render prior predictions obsolete. Within this framework, vascular access timing becomes an iterative, patient-centered process rather than a discrete decision anchored to a calendar date. A patient with a 25%–30% predicted 2-year risk of kidney failure but a high competing risk of death or loss of function may derive limited benefit from early AVF creation. By contrast, a patient with rapidly progressive disease, low competing risk, and a strong preference for dialysis preparedness may reasonably favor earlier intervention despite uncertainty in exact timing.

**Figure 1:**
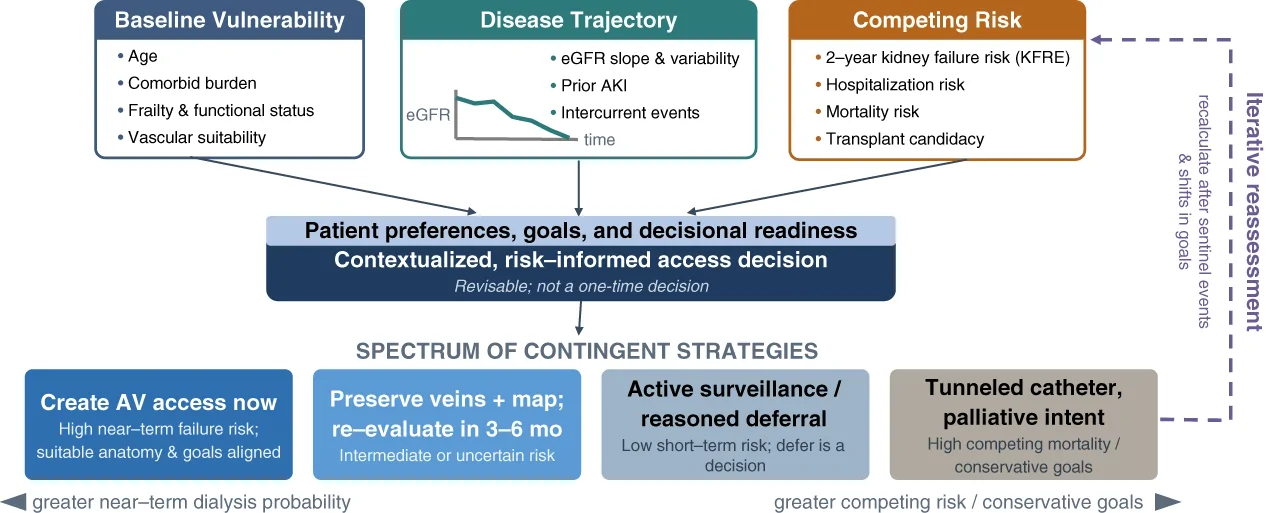
**Beyond the clock: dynamic, risk-informed vascular access planning in older adults.** Access decisions emerge from the convergence of evolving clinical vulnerability, disease trajectory, near-term kidney failure risk, and patient goals rather than from a single calendar date or eGFR threshold. KFRE, Kidney Failure Risk Equation.

It can be concluded that the notion of a singular right time for AVF creation is misguided. In an aging CKD population, access timing is often a moving target, shaped by changing disease trajectory, competing risks, and patient priorities. The more meaningful standard is not whether access was created within a discrete interval, but whether the decision was sound for that patient at that moment. The work by Heggen *et al*. lends support to this broader view of vascular access planning as a revisable, patient-centered process rather than one judged by a rigid temporal benchmark alone.

## Supplementary Material
