# Supplementary material for: Vascular Access in Older Adults Approaching Kidney Failure: Is There a Single Right Time?
Source: Kidney360. 2026 Sep 24;7(9):1918–20. doi: 10.34067/KID.0000001373 (PMC13641442; doi:10.34067/KID.0000001373)
Supplement: Supplementary file 1 [file kidney360-7-1918-s001.pdf]

ASN American Society of Nephrology
JASN Journal of the American Society of Nephrology
KIDNEY360 Accessing Our World From Every Angle
CJASN Clinical Journal of the American Society of Nephrology

ASN Journal Disclosure Form

As per ASN journal policy, I have disclosed any financial relationships or commitments I have held in the past 36 months as included below. I have listed my Current Employer below to indicate there is a relationship requiring disclosure. If no relationship exists, my Current Employer is not listed.

M. Murea has nothing to disclose.

I understand that the information above will be published within the journal article, if accepted, and that failure to comply and/or to accurately and completely report the potential financial conflicts of interest could lead to the following: 1) Prior to publication, article rejection, or 2) Post-publication, sanctions ranging from, but not limited to, issuing a correction, reporting the inaccurate information to the authors' institution, banning authors from submitting work to ASN journals for varying lengths of time, and/or retraction of the published work.

Name: Mariana Murea
Manuscript ID: K360-2026-000910
Manuscript Title: Vascular Access in Older Adults Approaching Kidney Failure: Is There a Single "Right" Time?
Date of Completion: July 21, 2026
Disclosure Updated Date: April 16, 2026

ASN American Society of Nephrology
JASN Journal of the American Society of Nephrology
KIDNEY360 Accessing Our World From Every Angle
CJASN Clinical Journal of the American Society of Nephrology

ASN Journal Disclosure Form

As per ASN journal policy, I have disclosed any financial relationships or commitments I have held in the past 36 months as included below. I have listed my Current Employer below to indicate there is a relationship requiring disclosure. If no relationship exists, my Current Employer is not listed.

T. Robinson has nothing to disclose.

I understand that the information above will be published within the journal article, if accepted, and that failure to comply and/or to accurately and completely report the potential financial conflicts of interest could lead to the following: 1) Prior to publication, article rejection, or 2) Post-publication, sanctions ranging from, but not limited to, issuing a correction, reporting the inaccurate information to the authors' institution, banning authors from submitting work to ASN journals for varying lengths of time, and/or retraction of the published work.

Name: Todd W. Robinson
Manuscript ID: K360-2026-000910
Manuscript Title: Vascular Access in Older Adults Approaching Kidney Failure: Is There a Single "Right" Time?
Date of Completion: July 21, 2026
Disclosure Updated Date: July 21, 2026
